# Soft and Stiff Simplex Tensegrity Lattices as Extreme Smart Metamaterials

**DOI:** 10.3390/ma12010187

**Published:** 2019-01-08

**Authors:** Anna Al Sabouni-Zawadzka, Wojciech Gilewski

**Affiliations:** Faculty of Civil Engineering, Warsaw University of Technology, 00-637 Warsaw, Poland; w.gilewski@il.pw.edu.pl

**Keywords:** metamaterial, tensegrity lattice, extreme material

## Abstract

The present paper is dedicated to an evaluation of novel cellular metamaterials based on a tensegrity pattern. The materials are constructed from supercells, each of which consists of a number of simplex modules with different geometrical proportions. Mechanical properties of the metamaterial can be controlled by adjusting the level of self-equilibrated forces or by changing the properties of structural members. A continuum model based on the equivalence of strain energy of the 3D theory of elasticity with a discrete formulation is used to identify the qualitative properties of the considered metamaterials. The model allows the inclusion of nonlinearities related to the equations of equilibrium in actual configuration of the structure with self-equilibrated set of normal forces typical for tensegrities. The lattices are recognised as extreme metamaterials according to the eigensolution of the equivalent elasticity matrices of the continuum model. The six representative deformation modes are defined and discussed: stiff, soft and medium extensional modes and high (double) as well as low shear modes. The lattices are identified as unimode or nearly bimode according to the classification of extreme materials.

## 1. Introduction

Identification of unusual mechanical properties of tensegrity structures is one of the interesting challenges of exploring the mechanics of engineering metamaterials. Metamaterials are usually defined as man-made composites that do not exist in nature and have atypical or unusual properties [1,2]. In recent years, there have been many significant and important scientific studies in the field of metamaterials with unusual mechanical properties [2] such as: negative Poisson’s ratio, atypical dynamic characteristics, unusual volume changes and expansion modules, very light materials, ultra-stiff or ultra-soft materials. Smart materials are defined as those in which one form of energy (mechanical, magnetic, electrical, etc.) is transformed into another one in a reversible and repeatable way [3]. Smart structures are capable of diagnosing changes in the environment and reacting to them in an adaptive way [4]. These features distinguish them from typical structures, whose main purpose is to provide load capacity and to ensure the safety of people. At the same time, we observe the adaptability to functional needs, modifications of the shape of the structure, changes of stiffness or damping properties, in order to minimize deformation and possible damage. Taking into account the above definitions, a smart metamaterial should be regarded as a smart structure rather than as a smart material.

An interesting idea of modern research in this field is metamaterials based on origami patterns [5,6], which are inspired by the ancient art of paper folding. One of the most popular and effective patterns are the Miura-Ori folds. Similarly to the origami patterns, tensegrities seem to show a great potential for construction of metamaterials with non-typical mechanical characteristics [7,8,9]. For the purpose of the present work, tensegrities are defined as cable-strut systems with a special configuration of nodes. They are statically indeterminate structures in a stable equilibrium. Struts form a discontinuous system of members under compression that is surrounded by a continuous system of elements in tension, which exhibit no compressive stiffness. Particular features of tensegrity structures are infinitesimal mechanisms, balanced with self-stress states [10]. Occurrence of a self-stress state in a structure indicates that there is a certain set of internal forces in structural members, which are independent from external loading and boundary conditions because they are in self-equilibrium.

The main advantages of pin-joined tensegrity structures are: high stiffness-to-mass ratio, controllability, reliability and deployability [11,12]. Furthermore, tensegrities exhibit some special features, which are a result of the occurrence of infinitesimal mechanisms that are stabilized by the system of normal forces in self-equilibrium. One can control their static and dynamic characteristics with an adjustment of the pre-stressing forces [13,14]. 

As was presented in reference [11], there are some particular features of tensegrity structures following which one can classify them as smart structures. These features are: self-control, self-diagnosis, self-repair and self-adjustment (active control) with the use of self-stress as well as geometrical properties of the structure.

The term “tensegrity metamaterial” was first introduced in reference [15] for the dynamics of the chain of tensegrity prisms and developed in references [16,17]. A similar concept was analyzed in reference [18]. Self-similar tensegrity columns of order 1 and higher order were proposed in reference [19]. The paper reference [20] is dedicated to the morphological optimization of tensegrity-type metamaterials with a prototypical model of an infinite slab. The formulation of the novel class θ=1 units was presented in reference [21] with the discussion of potential of such structures for mechanical metamaterials. Geometrically nonlinear behavior of uniformly compressed tensegrity prisms with extreme softening/stiffening response is recognized in reference [22] with relation to the design and manufacture of tensegrity lattices and innovative metamaterials. An interesting method to construct 3D tensegrity lattices from truncated octahedron elementary cells was proposed and discussed in reference [23] and extended for phase transition in reference [24]. Various automatically assembled tensegrity lattices were proposed in reference [25] for large scale structures. Metal rubber was introduced into the struts of a tensegrity prisms in references [26,27]. Both the theoretical and experimental data show the significant improvement of energy absorption and tunable dynamic properties to create an efficient mechanical metamaterial. Smart properties as well as a negative Poisson’s ratio were observed in reference [28] for the orthotropic metamaterial based on the simplex tensegrity pattern. To conclude, great potential and dynamic development of the tensegrity based metamaterials have been observed in the literature of the last few years. 

The concept of extreme materials was introduced in reference [29]. They are defined as extremely stiff materials under the action of certain stresses, or extremely compliant in other orthogonal cases of stresses. Extreme materials often have negative Poisson’s ratios. The study of extreme properties of materials is based on the analysis of elasticity tensor. As is known, it must be positive definite and in the theory of elasticity it shows certain types of symmetries. This tensor can be diagonalized by orthogonal transformation. If we present the components of the elastic tensor in the Voight’s form as a square matrix **E** of dimensions 6 × 6, its diagonal representation is the set of eigenvalues $\lambda_{i}>0$ (*i* = 1, 2, …, 6), and orthogonal eigenvectors $w_{i}$ describe the appropriate forms of deformation. One can classify materials as nullmode, unimode, bimode, trimode, quadramode, pentamode or hexamode [29,30] depending on the number of eigenvalues $\lambda_{i}$ that are very small. Such a classification is often used in determining the properties of metamaterials, as long as the elastic matrix **E** is known. 

The present paper is dedicated to the analysis of the tensegrity inspired metamaterials in view of possible extreme properties. The continuum model of mechanical properties of tensegrity lattices is used to define the equivalent elasticity matrix **E**. The proposed continuum model [28,31] is applied to identify the extreme properties of the proposed metamaterial. Its extreme characteristics can be controlled with the self-stress state and cable to strut properties ratio, following the features of smart metamaterials. Three representative tensegrity lattices based on the 4-strut simplex module are discussed. According to the best knowledge of the authors there are no papers in this field in the available literature.

## 2. Continuum Model of a Tensegrity Lattice

The continuum model is based on the comparison of the strain energy of a tensegrity structure defined using a discrete model and the strain energy of a solid determined according to the symmetric 3D elasticity theory [28,31]. 

A discrete model describes a tensegrity pin-joined truss structure which is composed of *e* straight and prismatic bars of the lengths $l_{k}$, cross sections $A_{k}$ and Young’s modulus $E_{k}$. The bars are connected in nodes in which a number of s nodal displacements $q_{j}$ and nodal forces $Q_{j}$ are defined (see [32,33] for details). Axial forces $N_{k}$ can be expressed by the extensions of bars $\Delta_{k}$ in the form $N_{k}=E_{k}A_{k}\Delta_{k}/l_{k}$. The extensions $\Delta_{k}$ are a combination of nodal displacements $\Delta_{k}=\sum_{j=1}^{s}B_{kj}q_{j}$, *J* = 1,2,…,*s*. *B_kj_* is a compatibility matrix of projection of nodal displacements for the directions of bar axes [32,33]. Additionally, the self-equilibrated system of axial forces $S_{k}$, which satisfy the homogeneous set of equilibrium equations $\sum_{k=1}^{e}B_{jk}S_{k}=0$, is considered. If one considers the equations of equilibrium in the actual configuration, the moment $M_{k}=S_{k}l_{k}\psi_{k}$ is acting on each bar. Angles of bar rotations $\psi_{k}$ can be expressed as a combination of nodal displacements $\psi_{k}=\frac{1}{l_{k}}\sum_{j=1}^{s}C_{kj}q_{j}$. *C_kj_* is an algebraic matrix of a projection of nodal displacements for the directions perpendicular to bar axes [34]. The above formalism leads to the linear system of algebraic equations $\sum_{j=1}^{s}(k_{ij}+k_{ij}^{G})q_{j}=Q_{i}$, in which the linear stiffness matrix $k_{ij}$ and geometric stiffness matrix $k_{ij}^{G}$ can be expressed in algebraic form $k_{ij}=\sum_{k=1}^{e}B_{ki}\frac{E_{k}A_{k}}{l_{k}}B_{kj}$, $k_{ij}^{G}=\sum_{k=1}^{e}C_{ki}\frac{S_{k}}{l_{k}}C_{kj}$ (see references [33,34] for further details). The approach is not dependent on any approximation typical for the finite element method.

In a discrete model (DM), the strain energy of a tensegrity truss can be expressed in the matrix notation as a quadratic form of nodal displacements **q**:

(1) $$E_{s}^{\mathrm{DM}}=\frac{1}{2}q^{T}Kq,$$

where: $K=K_{L}+K_{G}$, $K_{L}$-global linear stiffness matrix, $K_{G}$-global geometric stiffness matrix.

The self-equilibrated system of axial forces of the structure is represented by the geometric stiffness matrix and is related to the equations of equilibrium in the actual configuration of the lattice.

The strain energy of a solid according to the symmetric geometrically linear 3D elasticity theory (ET) [35] can be expressed as:

(2) $$E_{s}^{\mathrm{ET}}=\frac{1}{2}\int_{V}\varepsilon^{T}E\varepsilon \;dV,$$

where: ε-vector of strain components, E-elasticity matrix.

**Figure 1:**
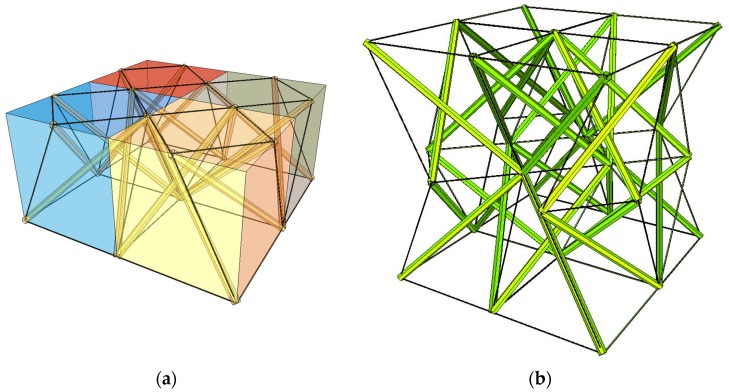
Geometry of a regular supercell (R): (**a**) A four-module supercell; (**b**) An eight module supercell [28].

In order to analyze mechanical properties of the material, it is proposed to compare the strain energy of an unsupported tensegrity to the strain energy of a cube, with an assumption that the strain energy of the cube is constant in its volume. In a general case both the analyzed structure and the solid can have arbitrary dimensions. However, in order to show how the continuum model is constructed, a typical tensegrity module inscribed into a cube of edge length a is considered (presented in Figure 1 of reference [28]).

With the above assumptions, the strain energy of the cube of edge length a, according to the symmetric 3D elasticity theory (ET):

(3) $$E_{s}^{\mathrm{ET}}=\frac{1}{2}\int_{V}\varepsilon^{T}E\varepsilon \;dV=\frac{1}{2}\varepsilon^{T}E\varepsilon \;a^{3}.$$

To compare the energies and build the equivalent elasticity matrix, the nodal displacements of the structure are expressed by the average mid-values of displacements and their derivatives in the center of the cube of edge length a, with the use of Taylor series expansion. Nodal coordinates of the analyzed tensegrity structure can be expressed using the parameter a, which corresponds to the edge length of the cube: $\left\{\alpha_{xi}a,\alpha_{yi}a,\alpha_{zi}a\right\}$. Then, the parameters of the node *i* (for example nodal displacements) can be described as:$\Delta x_{i}=\alpha_{xi}a$, $\Delta y_{i}=\alpha_{yi}a$, $\Delta z_{i}=\alpha_{zi}a$.

The next step of the analysis is a substitution of the determined nodal displacements in the formula (1). As a result, an expression containing a constant part (independent of a) and terms with a factor $a^{n}$, *n* ∈ {1, 2, …} is obtained. However, in the case of small values of a, terms with the factor $a^{n}$ can be regarded as higher order terms in the Taylor series expansion and should be omitted. Small values of the module dimension a should be considered as relative to the total dimensions of the metamaterial. Moreover, the mentioned terms contain displacement derivatives greater than the first one, which is beyond the scope of the symmetric theory of elasticity. 

Comparison of strain energies (1) and (3) leads to the determination of coefficients of the matrix **E**. In a general case of an anisotropic structure, the obtained elasticity matrix in Voight’s notation [32] has the following form:

(4) $$E=\left[\begin{array}{cccccc} e_{11} & e_{12} & e_{13} & e_{14} & e_{15} & e_{16}\\ & e_{22} & e_{23} & e_{24} & e_{25} & e_{26}\\ & & e_{33} & e_{34} & e_{35} & e_{36}\\ & & & e_{44} & e_{45} & e_{46}\\ & & & & e_{55} & e_{56}\\ sym. & & & & & e_{66} \end{array}\right].$$

It contains 36 coefficients, including 21 independent ones. The above matrix can take different and particular forms, with the type depending on eight possible symmetries [36]. The proposed continuum model is non-linear in the sense of equations of equilibrium considered in actual configuration. Numerical simulation and validation of the proposed continuum model is presented in the Appendix at the end of the present paper. Other possible continuum models of the lattices are discussed in references [37,38].

## 3. Simplex-Based Tensegrity Lattices 

The metamaterial considered in this paper is constructed from one of the most popular tensegrity modules—a 4-strut simplex (see references [7,9,28] for geometrical details). It is a typical tensegrity, which consists of four separate struts surrounded by the continuous system of twelve cables [7,8]. The 4-strut simplex module is obtained from a regular prism by rotating one of its bases 135 degrees clockwise or counter clockwise. 

One of the special characteristics of tensegrity structures are infinitesimal mechanisms that are balanced with a self-equilibrated system of normal forces [7,8,10]. The considered simplex module has one infinitesimal mechanism and one corresponding self-stress state—self-stress is expressed by the relative forces in struts and cables with a multiplier S_0_ (see reference [28] for the detailed description). 

It should be noticed that the proposed unit cell is an anisotropic structure. However, it is proved below that the metamaterial based on such unit cells exhibits orthotropic properties.

Simplex tensegrity modules described above can be arranged in different patterns to form a material with certain properties. Depending on the type of the module used (with the basis rotated clockwise or counter clockwise) and the way in which the modules are connected, a material with different mechanical characteristics can be obtained. In the present paper a material with orthotropic properties is proposed, as it exhibits some special features (negative Poisson’s ratio as an example [28]). 

A system that consists of four simplex modules joined together by common cables of the lower bases and common nodes of the upper bases is presented in Figure 1a. The modules are arranged alternately: a module that is rotated clockwise is put next to the module with the counter-clockwise rotation. Although a single simplex module is anisotropic, the whole structure has orthotropic properties. Following this method, a regular eight-module supercell (Figure 1b [28]), constructed using two four-module layers, was considered. The upper layer of the supercell was built from the four-module layer turned upside-down and connected with the bottom layer using common cables.

The elasticity matrix $E_{R}$ obtained from the continuum model of the considered regular supercell has the following form:

(5) $$E_{R}=\left[\begin{array}{cccccc} e_{R11} & e_{R12} & e_{R13} & 0 & 0 & 0\\ & e_{R11} & e_{R13} & 0 & 0 & 0\\ & & e_{R33} & 0 & 0 & 0\\ & & & e_{R12} & 0 & 0\\ & & & & e_{R13} & 0\\ sym. & & & & & e_{R13} \end{array}\right],$$

with the coefficients [28]:

$$\begin{array}{l} e_{R11}=\frac{2EA}{a^{2}}\left(0.314815+0.960318\cdot k-0.0794978\cdot \sigma \right),\\ e_{R12}=\frac{EA}{a^{2}}\left(0.2962963+0.353553\cdot k-0.0134742\cdot \sigma \right),\\ e_{R13}=\frac{EA}{a^{2}}\left(0.740741+0.268328\cdot k+0.17247\cdot \sigma \right),\\ e_{R33}=\frac{2EA}{a^{2}}\left(0.592593+1.07331\cdot k-0.17247\cdot \sigma \right), \end{array}$$

where: $k=\frac{{\left(EA\right)}_{cable}}{{\left(EA\right)}_{strut}}\;$, ${\left(EA\right)}_{strut}=EA$, $\sigma =\frac{S}{EA}$.

The volume of the regular lattice is $V_{R}=aA_{strut}\left(48.00+62.15k\right)$.

**Figure 2:**
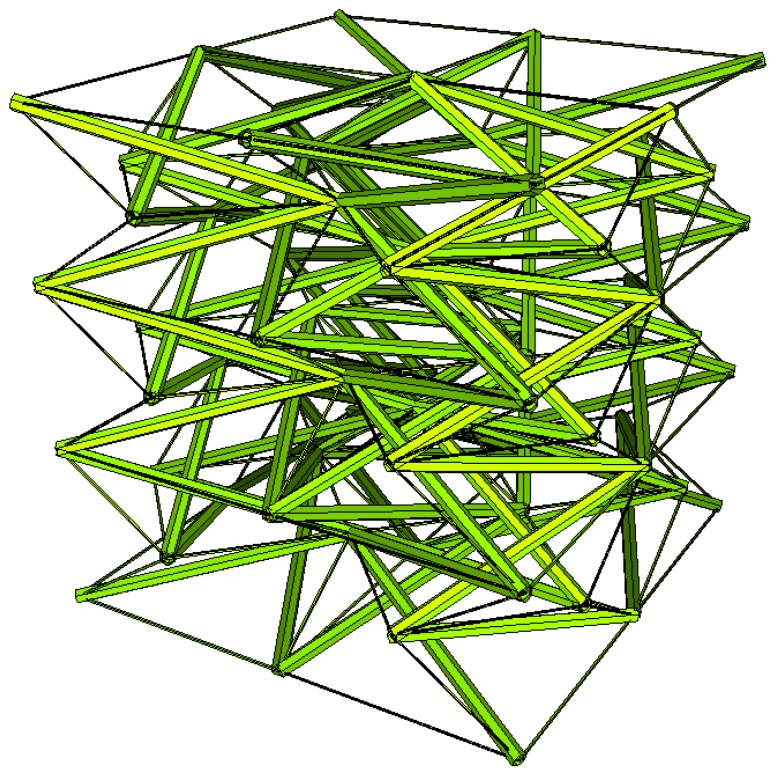
Tensegrity lattice with low modules (L).

**Figure 3:**
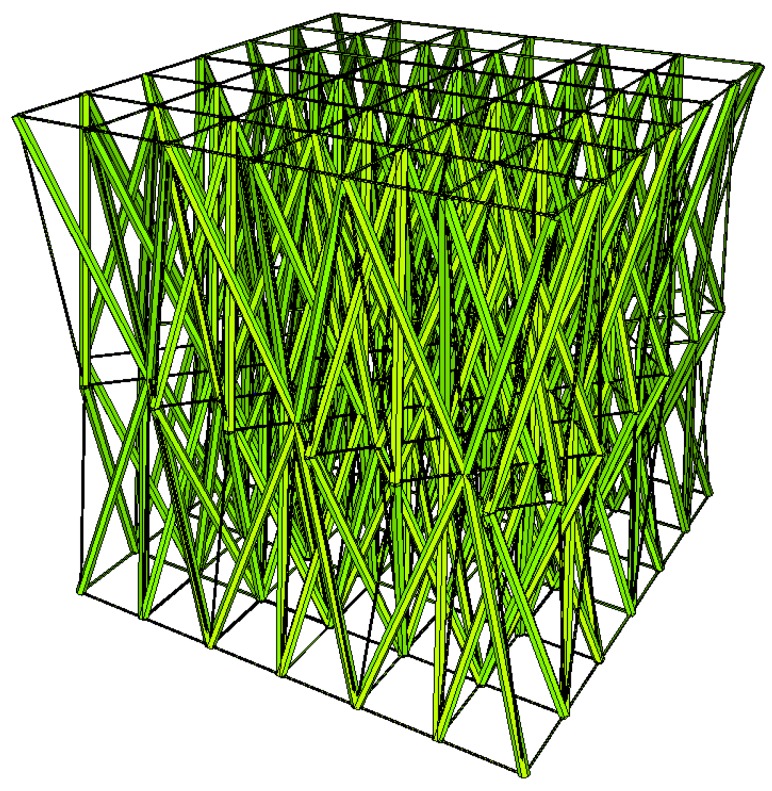
Tensegrity lattice with high modules (H).

Two other tensegrity lattices inscribed into a cube 2a×2a×2a are considered: with a small and large height-to-base area ratio of the module (Figure 2 and Figure 3).

Self-equilibrated forces for (L) and (H) models differ from the ones presented in Figure 1. The elasticity matrices $E_{L}$ and $E_{H}$ have orthotropic structures as before, but with different coefficients:

$$\begin{array}{l} e_{L11}=\frac{2EA}{a^{2}}\left(2.00729+2.67837\cdot k-0.135911\cdot \sigma \right),\\ e_{L12}=\frac{EA}{a^{2}}\left(1.88921+1.06066\cdot k+0.223898\cdot \sigma \right),\\ e_{L13}=\frac{EA}{a^{2}}\left(0.524781+0.576035\cdot k+0.0479246\cdot \sigma \right),\\ e_{L33}=\frac{2EA}{a^{2}}\left(0.0466472+0.256015\cdot k-0.0479246\cdot \sigma \right), \end{array}$$

with the volume $V_{L}=aA_{strut}\left(112.00+125.21k\right)$ and

$$\begin{array}{l} e_{H11}=\frac{2EA}{a^{2}}\left(0.0971325+2.28422\cdot k-0.217699\cdot \sigma \right),\\ e_{H12}=\frac{EA}{a^{2}}\left(0.0914188+1.06066\cdot k-0.134146\cdot \sigma \right),\\ e_{H13}=\frac{EA}{a^{2}}\left(2.05692+0.279923\cdot k+0.569545\cdot \sigma \right),\\ e_{H33}=\frac{2EA}{a^{2}}\left(14.8098+10.0772\cdot k-0.569545\cdot \sigma \right), \end{array}$$

with the volume $V_{H}=aA_{strut}=307.35+260.26k$.

The supercells are representative because the mechanical properties described in elasticity matrices do not change in repetitive bigger volumes. 

The proposed tensegrity metameterials (L), (R) and (H) will be compared and analyzed in the next chapter according to the eigenvalues and eigenvectors of the elasticity matrices under the assumption of an equal total volume.

## 4. Extreme Properties of Tensegrity Lattices

The eigenvalues $\lambda_{i}>0$ (*i* = 1, 2, …, 6), and orthogonal eigenvectors $w_{i}$ of elasticity matrices of the proposed metamaterials depend on the parameters k and σ. The eigenvalues in this chapter are calculated for the (L) and (H) elasticity matrices calibrated to an even volume of the material in each lattice (as in the metamaterial (R)). 

**Figure 4:**
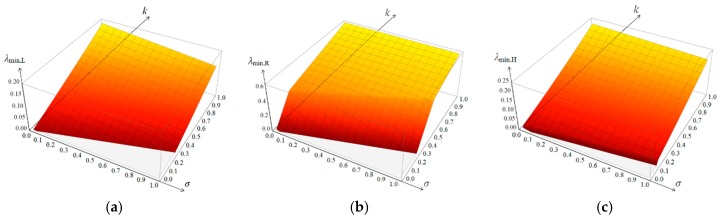
Distributions of lowest eigenvalue for: (**a**) Low; (**b**) Regular; (**c**) High metamaterial.

Let us start the analysis from the lowest eigenvalues. The elasticity matrices are positive definite so only positive eigenvalues are to be considered. The values of $\lambda_{\min}$ for k∈(0,1) and σ∈(0,1) for low (L), regular (R) and high (H) metamaterials are presented in Figure 4. 

**Figure 5:**
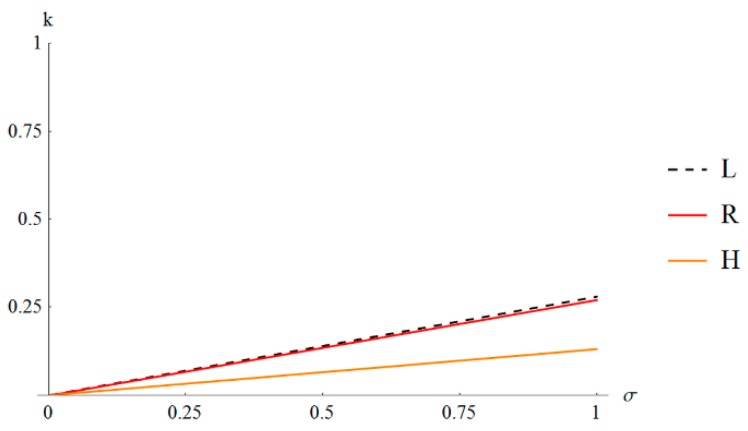
Lines of zero value of the lowest eigenvalue.

In the (L) metamaterial (Figure 4a) the lowest eigenvalue does not change the mode of deformation within the considered area. The metamaterial is unimode according to the classification of reference [29]. In the (R) metamaterial (Figure 4b) the change of the mode of deformation of the lowest eigenvalue is observed but far from the zero line. The material is also unimode for selected parameters. In the (H) metamaterial (Figure 4c) the change of the mode of deformation of the lowest eigenvalue is observed close to the zero line. It means that the material is close to bimode [29]. The lines of zero values representative for unimode soft deformation are presented in Figure 5 for all metamaterials together.

The highest eigenvalues of the matrices **E** depend on the parameters k and σ to a small extent (a couple of percent), therefore the analysis is not considered in this paper. 

**Figure 6:**
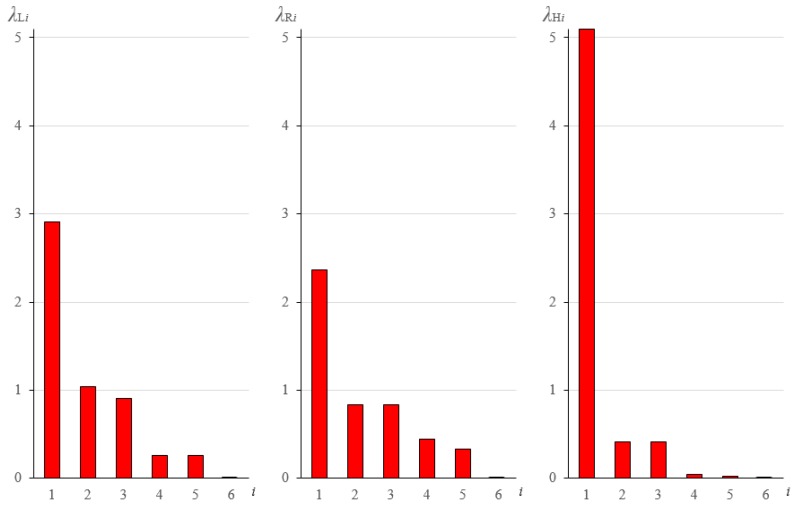
Distribution of eigenvalues for (L), (R) and (H) metamaterials.

The second part of the analysis is proposed for the parameter k=0.1 which is typical for standard cable-strut structures. The values of self-stress parameters necessary to obtain the zero lowest eigenvalue are then the following: $\sigma_{L}=0.35856$, $\sigma_{M}=0.37317$ and $\sigma_{H}=0.76479$. The sets of six eigenvalues are presented in Figure 6.

As it can be noticed, the most extreme metamaterial is (H). One eigenvalue is high, three are relatively small and two are close to zero. The (H) metamaterial is nearly bimode. The materials (L) and (R) can be defined as unimode. The sequence of eigenvectors in the (L) metamaterial is different than in the others—the double eigenvalue which is responsible for shear vertical deformation is next to the zero eigenvalue in the material (L) and next to the highest eigenvalue in the materials (R) and (H). Six deformation modes can be defined in the metamaterials: stiff (extensional), soft (extensional), named “easy” in [29], medium extensional, high shear (double) and low shear. The results are presented graphically in Table 1, Table 2, Table 3, Table 4 and Table 5.

The analysis carried out above for the parameter k=0.1 differs for other k values. A similar analysis can be performed on the basis of the matrix inverse to **E**, defining others that can be called stress modes instead of deformation modes.

## 5. Conclusions

The present paper focuses on the analysis of a novel cellular metamaterial based on the simplex tensegrity pattern recognized as a metamaterial. Three tensegrity lattices are proposed, which differ in geometrical proportions. The authors use a continuum model of the lattices to estimate the influence of self-equilibrated normal forces and geometrical parameters on the behavior of the system. Moreover, a spectral analysis of the elasticity matrices, with the condition of the equal total volume of the lattices, is presented as a tool for comparing the metamaterials and defining their extreme properties. 

The performed analyses allowed the authors to identify six typical deformation modes in the lattices, which were named stiff, soft and medium extensional modes, as well as high (double) and low shear modes. The proposed novel simplex based metamaterials can be regarded as extreme materials with unimode or nearly bimode mechanical properties. The occurrence of soft (easy) as well as stiff deformation modes allows for classifying the developed materials as smart tensegrity metamaterials, which is very promising as far as the engineering applications are concerned.

## Figures and Tables

**Table 1:** Stiff (extensional) mode of deformation.

$\lambda_{1L}=2.90631$	$\lambda_{1R}=2.36011$	$\lambda_{1H}=5.09819$
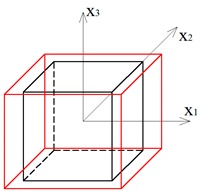	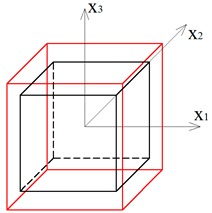	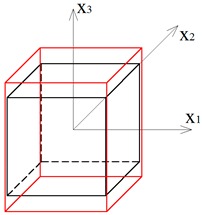
$w_{1L}=\left[\begin{array}{c} 5.444189\\ 5.444189\\ 1\\ 0\\ 0\\ 0 \end{array}\right]$	$w_{1R}=\left[\begin{array}{c} 0.654487\\ 0.654487\\ 1\\ 0\\ 0\\ 0 \end{array}\right]$	$w_{1H}=\left[\begin{array}{c} 0.0819302\\ 0.0819302\\ 1\\ 0\\ 0\\ 0 \end{array}\right]$

**Table 2:** Soft (extensional) mode of deformation.

$\lambda_{6L}\sim 0$	$\lambda_{6R}\sim 0$	$\lambda_{6H}\sim 0$
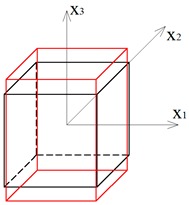	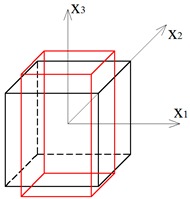	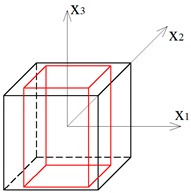
$w_{6L}=\left[\begin{array}{c} -0.0918408\\ -0.0918408\\ 1\\ 0\\ 0\\ 0 \end{array}\right]$	$w_{6R}=\left[\begin{array}{c} -0.763957\\ -0.763957\\ 1\\ 0\\ 0\\ 0 \end{array}\right]$	$w_{6H}=\left[\begin{array}{c} -1\\ -1\\ 0.16386\\ 0\\ 0\\ 0 \end{array}\right]$

**Table 3:** Medium extensional mode of deformation.

$\lambda_{2L}=1.04074$	$\lambda_{4R}=0.435737$		$\lambda_{4H}=0.0365033$
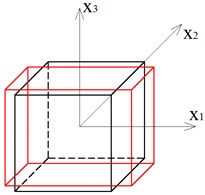		$w_{2L}=w_{4R}=w_{4H}=\left[\begin{array}{c} 1\\ -1\\ 0\\ 0\\ 0\\ 0 \end{array}\right]$	

**Table 4:** High shear modes of deformation.

$\lambda_{4L}=\lambda_{5L}=0.26249$	$\lambda_{2R}=\lambda_{3R}=0.831934$		$\lambda_{2H}=\lambda_{3H}=0.412162$
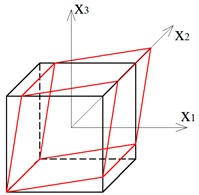		$w_{4L}=w_{2R}=w_{2H}=\left[\begin{array}{c} 0\\ 0\\ 0\\ 0\\ 1\\ 0 \end{array}\right]$	
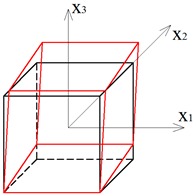		$w_{5L}=w_{3R}=w_{3H}=\left[\begin{array}{c} 0\\ 0\\ 0\\ 0\\ 0\\ 1 \end{array}\right]$	

**Table 5:** Low shear mode of deformation.

$\lambda_{3L}=0.908675$	$\lambda_{5R}=0.326623$		$\lambda_{5H}=0.015517$
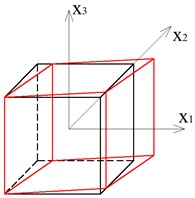		$w_{5L}=w_{5R}=w_{5H}=\left[\begin{array}{c} 0\\ 0\\ 0\\ 1\\ 0\\ 0 \end{array}\right]$	

**Figure A1:**
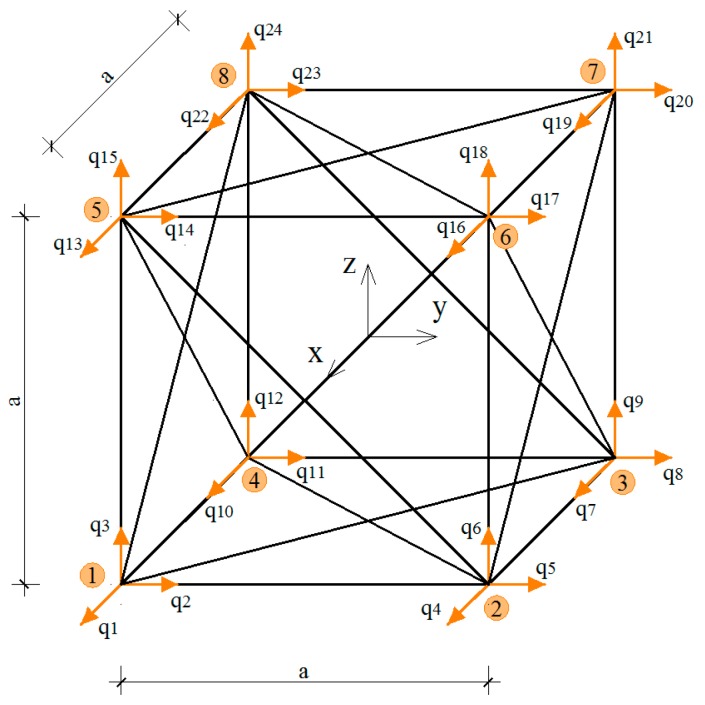
Space truss with 8 nodes and 24 degrees-of-freedom.

**Figure A2:**
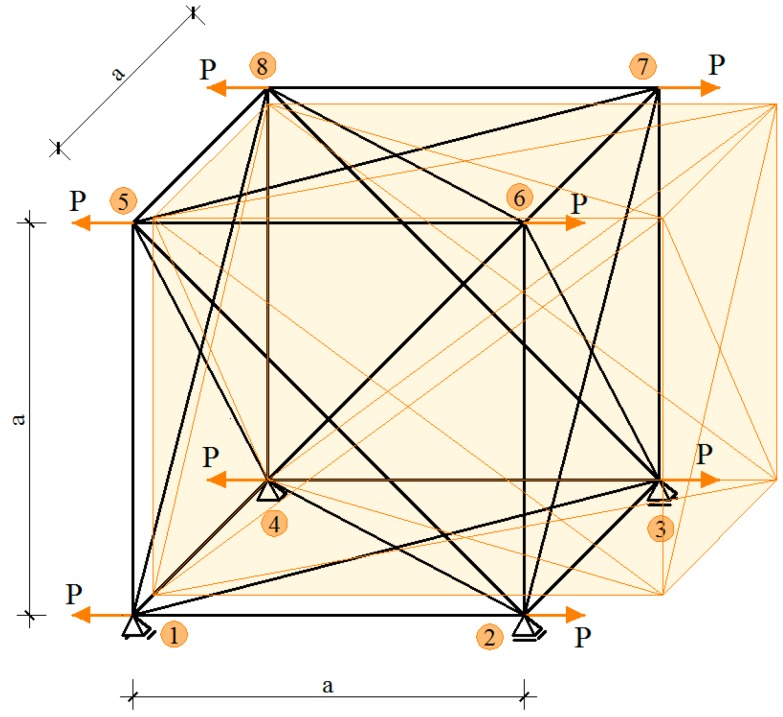
State *σ*_y_ = *const*—a deformed structure

## Appendix A. Validation of the Continuum Model

In order to verify the proposed continuum model, its validation on an orthotropic structure was performed. The structure that was chosen for the verification of the model has a simple geometry and therefore, its behavior is easy to understand. The results obtained from the continuum model were compared with the ones from the discrete analysis. The calculations were made in the *Mathematica* software. The validation was performed on a space truss (Figure A1) shaped in such a way that it is an orthotropic structure. The orthotropy was obtained through diversification of the stiffness of particular truss members: members parallel to the axis *x*—stiffness *EA*, members parallel to the axis *y*—stiffness 2*EA*, members parallel to the axis *z*—stiffness 3*EA*, diagonal members—stiffness *EA*.

To validate the model, the following mechanical characteristics were compared: Young’s moduli, Poisson’s ratios and shear moduli. In the validation a geometric stiffness matrix representing the self-stress of the truss was considered. In order to simplify the calculations, so that the results could easily be compared, a certain level of self-stress *σ* = *S*/*EA* = 0.5 was assumed.

In the continuum analysis the structure was not supported, as it is not required in the proposed model. The following elasticity matrix **E** was obtained for the analyzed truss using the continuum model with self-stress included:

(A1) $$E=\frac{EA}{a^{2}}\left[\begin{array}{cccccc} 2\times \left(2+\sqrt{2}+2\sigma \right) & \sqrt{2}-2\sigma & \sqrt{2}-2\sigma & 0 & 0 & 0\\ & 2\times \left(4+\sqrt{2}+2\sigma \right) & \sqrt{2}-2\sigma & 0 & 0 & 0\\ & & 2\times \left(6+\sqrt{2}+2\sigma \right) & 0 & 0 & 0\\ & & & \sqrt{2}+14\sigma & 0 & 0\\ & & & & \sqrt{2}+14\sigma & 0\\ sym. & & & & & \sqrt{2}+14\sigma \end{array}\right].$$

The inverse matrix **H** = **E**^−1^, with the assumption *σ* = 0.5 is the following:

(A2) $$H=\frac{a^{2}}{EA}\left[\begin{array}{cccccc} 0.113565 & -0.00357947 & -0.00270719 & 0 & 0 & 0\\ -0.00357947 & 0.0781267 & -0.0018349 & 0 & 0 & 0\\ -0.00270719 & -0.0018349 & 0.0595351 & 0 & 0 & 0\\ 0 & 0 & 0 & 0.118847 & 0 & 0\\ 0 & 0 & 0 & 0 & 0.118847 & 0\\ 0 & 0 & 0 & 0 & 0 & 0.118847 \end{array}\right].$$

Mechanical characteristics were calculated from the matrix **H** (A2), using the relations described in terms of mechanical coefficients of orthotropy [28,35]. For example, the analysis of a state *σ*_y_ = *const* leads to the determination of: Young’s modulus *E*_2_ and Poisson’s ratios *ν*_21_ and *ν*_23_ in the following way:

(A3) $$\frac{1}{E_{2}}=0.0781267\frac{a^{2}}{EA}\;\Rightarrow \;E_{2}=12.7997\;\frac{EA}{a^{2}},$$

(A4) $$-\frac{\nu_{21}}{E_{2}}=-0.00357947\frac{a^{2}}{EA}\;\Rightarrow \;\nu_{21}=0.04582,$$

(A5) $$-\frac{\nu_{23}}{E_{2}}=-0.0018349\frac{a^{2}}{EA}\;\Rightarrow \;\nu_{23}=0.02349,$$

In the same way, all other mechanical characteristics can be determined by analysing different stress states. The following values of mechanical properties were obtained:

(A6) $$\begin{array}{l} E_{1}=8.8055\;\frac{EA}{a^{2}},\\ E_{2}=12.7997\;\frac{EA}{a^{2}},\\ E_{3}=16.7968\;\frac{EA}{a^{2}}, \end{array}\;\begin{array}{l} G_{1}=8.41421\;\frac{EA}{a^{2}},\\ G_{2}=8.41421\;\frac{EA}{a^{2}},\\ G_{3}=8.41421\;\frac{EA}{a^{2}}, \end{array}\;\begin{array}{l} \nu_{12}=0.03152,\\ \nu_{13}=0.02384,\\ \nu_{21}=0.04582,\\ \nu_{23}=0.02349,\\ \nu_{31}=0.04547,\\ \nu_{32}=0.03082. \end{array}$$

Six typical stress states were analyzed using the discrete model. In each state the structure was supported in nodes in such a way that proper deformations could occur. The applied load caused in each case the stress of a unit value. The concentrated forces $P=\frac{1\cdot a^{2}}{4}$ were applied to the proper nodes of the truss. For all analyzed stress states nodal displacements of the structure were determined and mechanical characteristics were calculated. In the analysis self-stress of the truss on the level of *σ* = 0.5 was applied.

Figure A2 shows the deformed truss in one of the analyzed states: *σ*_y_ = *const*, which corresponds to the uniform tension in the direction *y.* The adopted support scheme enables a free deformation of the structure in the considered uniform tension state.

The following values of nodal displacements were obtained in the analyzed state (the above values are divided by the factor *a*^3^/*EA*):

(A7) $$\begin{array}{l} q_{1}=-0.00358,\\ q_{4}=-0.00358,\\ q_{7}=0,\\ q_{10}=0,\\ q_{13}=-0.00358,\\ q_{16}=-0.00358,\\ q_{19}=0,\\ q_{22}=0, \end{array}\;\begin{array}{l} q_{2}=0,\\ q_{5}=0.07813,\\ q_{8}=0.07813,\\ q_{11}=0,\\ q_{14}=0,\\ q_{17}=0.07813,\\ q_{20}=0.07813,\\ q_{23}=0, \end{array}\;\begin{array}{l} q_{3}=0,\\ q_{6}=0,\\ q_{9}=0,\\ q_{12}=0,\\ q_{15}=-0.00183,\\ q_{18}=-0.00183,\\ q_{21}=-0.00183,\\ q_{24}=-0.00183. \end{array}$$

The presented state *σ*_y_ = *const* was used to determine the values of: Young’s modulus *E*_2_ and Poisson’s ratios *ν*_21_ and *ν*_23_ in the following way:

(A8) $$\begin{array}{l} E_{2}=\frac{\sigma_{y}}{\varepsilon_{y}}=\frac{1\cdot a}{q_{5}}=\frac{1\cdot a}{0.07813}\cdot \frac{EA}{a^{3}}=12.7997\;\frac{EA}{a^{2}},\\ \nu_{21}=-\frac{\varepsilon_{x}}{\varepsilon_{y}}=-\frac{q_{1}}{q_{5}}=-\frac{-0.00358}{0.07813}=0.04582,\\ \nu_{23}=-\frac{\varepsilon_{z}}{\varepsilon_{y}}=-\frac{q_{15}}{q_{5}}=-\frac{-0.00183}{0.07813}=0.02349. \end{array}$$

It should be noticed that the above results are equal to the ones from the continuum model. Similar calculations were performed for other five stress states, in each case the results obtained from both analyses were fully consistent.

The validation of the proposed continuum model performed on a simple truss proved that it gives reliable results and therefore, it can be used to analyze extreme properties of various types of structures with self-stress.
